# Cognitive deficits are associated with poorer simulated driving in older adults with heart failure

**DOI:** 10.1186/1471-2318-13-58

**Published:** 2013-06-12

**Authors:** Michael L Alosco, Mary Beth Spitznagel, Mary Jo Cleveland, John Gunstad

**Affiliations:** 1 Department of Psychology, Kent State University, Kent, OH, USA; 2 Center for Senior Health, Summa Health System, Akron, OH, USA

**Keywords:** Driving simulation, Heart failure, Cognitive function, Driving ability

## Abstract

**Background:**

Cognitive impairment is prevalent in older adults with heart failure (HF) and associated with reduced functional independence. HF patients appear at risk for reduced driving ability, as past work in other medical samples has shown cognitive dysfunction to be an important contributor to driving performance. The current study examined whether cognitive dysfunction was independently associated with reduced driving simulation performance in a sample of HF patients.

**Methods:**

18 persons with HF (67.72; SD = 8.56 year) completed echocardiogram and a brief neuropsychological test battery assessing global cognitive function, attention/executive function, memory and motor function. All participants then completed the Kent Multidimensional Assessment Driving Simulation (K-MADS), a driving simulator scenario with good psychometric properties.

**Results:**

The sample exhibited an average Mini Mental State Examination (MMSE) score of 27.83 (SD = 2.09). Independent sample *t*-tests showed that HF patients performed worse than healthy adults on the driving simulation scenario. Finally, partial correlations showed worse attention/executive and motor function were independently associated with poorer driving simulation performance across several indices reflective of driving ability (i.e., centerline crossings, number of collisions, % of time over the speed limit, among others).

**Conclusion:**

The current findings showed that reduced cognitive function was associated with poor simulated driving performance in older adults with HF. If replicated using behind-the-wheel testing, HF patients may be at elevated risk for unsafe driving and routine driving evaluations in this population may be warranted.

## Background

Heart failure (HF) has become one of the most prevalent forms of cardiovascular disease with an estimated 600,000 persons newly diagnosed each year [1]. HF is the most common reason for recurrent hospitalizations [2] and is associated with elevated mortality risk [3] and reduced ability to perform key instrumental activities of daily living [4].

Recent work also shows that cognitive impairment in HF is associated with reduced driving independence [5]. Indeed, a history of heart disease has been previously linked with decreased driving amount and self-imposed driving cessation [6,7]. Cognitive impairment may underlie such findings, as on-road driving abilities require complex cognitive processes [8]. Consistent with this notion, cognitive impairment can be found in up to 75% of HF patients with frequent deficits exhibited in cognitive domains shown to be important contributors to driving ability in other medical populations (e.g., Alzheimer’s disease, mild cognitive impairment), including global cognitive function, attention, executive function, and psychomotor speed [9-16].

It is possible that reduced cognitive function in HF patients may impede their ability to perform complex driving behaviors (e.g., multitasking, traffic signal approach speed, and mean speed around curves [17]). Driving requires significant mental flexibility consisting of the cognitive ability to quickly process information and make decisions [15,18-20]. Past work that has examined neuropsychological test predictors of driving ability supports this notion. For instance, poorer performance on neuropsychological tests of attention and executive function is associated with greater risk of failing on-road driving tests and has also been shown to predict “at-risk” drivers among cognitively impaired populations [21,22].

Because of the prevalence of impairments in frontal systems function (e.g., attention/executive function/psychomotor speed) it would be expected that HF patients exhibit driving errors that involve organization, planning, and reasoning such as choosing the travel route and travel times, monitoring distance, adjusting speed, among other executive skills [17]. This pattern is distinct from driving deficits typically found in healthy elderly, which are primarily attributed to declines in perceptual ability and visuospatial deficits [23,24]. Moreover, reduced driving ability in HF may be predictable based on cerebrovascular changes, as cerebral hypoperfusion is thought to underlie cognitive impairment in this population and has also been identified as a contributor to greater driving impairments in Alzheimer disease patients [25]. Lastly, although cognitive status is a key predictor of driving ability [26,27], HF patients are also at also at risk for poor driving as result of factors associated with aging such as reduced visual acuity, hearing deficits, arthritis, bradykinesia, among others [28].

The above findings suggest that HF patients may be at risk for reduced driving ability, though no study to date has examined the relationship between cognitive function and driving performance among patients with HF. The current study sought to determine whether reduced neuropsychological test performance was associated with poorer driving performance using driving simulation technology in a sample of older adults with HF. To further evaluate risk of poor driving ability in this population, we also compared simulated driving performance between HF patients and healthy young adults.

## Methods

### Participants

A sample of 18 participants with HF was recruited from an ongoing NIH study examining neurocognitive function among older adults with HF. Strict inclusion and exclusion criteria were implemented in order to capture the independent effects of HF on neurocognitive outcomes. The inclusion criteria were age of 50-85 years, English as a primary language, and a diagnosis of New York Heart Association (NYHA) class II or III at the time of enrollment. Additional inclusion criteria also required a valid driver’s license and currently driving. Prior to study entry, all patients were screened for neurological and psychiatric conditions that may influence cognitive function or restrict their performance of instrumental activities of daily living. Specifically, individuals were precluded from study entry if they had a history of neurological disorders (e.g., dementia, stroke, multiple sclerosis, etc.), head injury with >10 minutes loss of consciousness, severe psychiatric disorder (e.g. schizophrenia, bipolar disorder), past or current substance abuse/dependence, and renal failure.

Participants averaged 67.72 (SD = 8.56) years of age, and were 11.1% female. Medical chart review also revealed that the sample had an average left ventricular ejection fraction of 39.38 (SD = 13.25). See Table 1 for demographic and medical information. Data from driving simulation performance of 97 healthy adults (19.91 (SD = 3.00) years of age, 54.6% female, 12.77 (SD = 1.19) years of education, and 87.6% Caucasian) that completed the simulation as part of a larger separate study was also used to compare HF patient performance to a healthy population [29].

**Table 1:** Demographic, cognitive, and driving simulation characteristics of 18 older adults with heart failure

**Demographic/medical characteristics**	
Age, mean (SD)	67.72 (8.56)
Years of education, mean (SD)	13.78 (2.13)
Female (%)	11.1
Race (% Caucasian)	100%
LVEF, mean (SD)	39.38 (13.25)
Hypertension (% yes)	72.2
History of myocardial infarction (% yes)	38.9
Diabetes (% yes)	38.9
Sleep apnea (% yes)	27.8
Cardiac medication status (% Angiotensin; % ACE; % beta-blocker; % diuretics)	27.8; 61.1; 55.6; 22.2
**Raw cognitive test performance, mean (SD)**	
*Global cognitive function*	
MMSE	27.83 (2.09)
*Attention/executive function*	
TMTA (seconds)	35.72 (11.21)
TMTB (seconds)	100.94 (56.51)
LNS	10.11 (2.85)
Stroop	31.17 (9.92)
Digit symbol coding	55.11 (13.06)
*Memory*	
CVLT Total	40.67 (9.05)
CVLT SDFR	8.17 (2.53)
CVLD LDFR	8.44 (3.20)
*Fine motor dexterity (seconds)*	
Pegboard dominant hand	88.00 (16.33)
Pegboard non-dominant hand	98.56 (20.62)
**Driving simulation performance, mean (SD)**	
Total collisions	2.00 (1.28)
Number of stop signs missed	.78 (.81)
Number of centerline crossings	5.83 (3.96)
Number of road excursions	4.83 (6.39)
% Over speed limit	9.06 (8.57)
% Out of lane	4.75 (3.16)

*LVEF* Left Ventricular Ejection Fraction, *MMSE* Mini Mental State Examination, *TMTA* Trail Making Test A, *TMTB* Trail Making Test B, *LNS* Letter Number Sequencing, *CVLT* California Verbal Learning Test-II, *SDFR* Short Delay Free Recall, *LDFR* Long Delay Free Recall, *Angiotensin* Angiotensin II Receptor Block, *ACE* Angiotensin-converting enzyme.

### Measures

#### Driving simulation

The STISIM Driving Simulator (Build 2.08.03) by Systems Technology Inc. was used to assess driving ability. It is a computer based, interactive drive simulator software package, and has been configured to control: (a) a high fidelity steering wheel with two analog levers for left/right turn indication; (b) an analog pedal set consisting of an analog brake pedal and another pedal for gas; and (c) a 46” High Definition LCD television at an average presentation distance of 4.5 feet. The STSIM driving software was used to devise the driving scenarios that all participants completed. The STSIM has been shown to correlate with on-road driving tests [30,31].

HF participants first completed a practice scenario with the driving simulation technology. This was conducted in order for drivers to become comfortable with the simulator and practice the tasks they will be required to perform during the test trial such as accelerating, decelerating, following the speed limit signs, navigating turns, stopping at stop signs/traffic signals, and maintaining lane position. The practice scenario is nearly 3 miles long, lasted approximately 15 minutes, and assessed driving performance in multiple settings, including city, country, and highway environments.

Participants then completed the Kent Multidimensional Assessment Driving Simulation (K-MADS) driving scenario, which is distinct from the practice simulation. All participants were instructed to drive as safe as possible as they normally would on the road. The K-MADS is a roughly 7 mile long driving scenario that takes approximately 20-25 minutes to complete. It has good psychometric properties (among an adult population, test-retest indices at two weeks range from r = 0.68 to r = 0.83; performance correlated with history of tickets in real world driving, r = 0.76) [29]. The K-MADS provides an opportunity to measure driving performance in a number of environments including a quiet suburb, a country road, a small town, and a busy city, each with its own speed limit restrictions and lane configurations. The course was designed to simulate a possible commute to school or work, such that individuals start in a quiet suburban neighborhood and drive on a highway to the nearby, medium-sized city. These same procedures were also performed in the healthy sample. For a full description and review of the K-MADS course refer to Alosco and colleagues (2012) [29].

The K-MADS yields several indices indicative of driving performance that were utilized in the current study, including total collisions, number of stop signs missed, number of centerline crossings, number of road excursions, % of time over the speed limit, and% of time out of the lane.

#### Neuropsychological measures

A brief battery of neuropsychological tests was administered to assess cognitive function across multiple domains. All neuropsychological tests used in the current study exhibit strong psychometric properties, including excellent reliability and validity. The domains and neuropsychological tests administered are as follows:

##### Global cognitive function

The Mini Mental State examination (MMSE) was used to assess global cognitive status. It is a widely used, brief cognitive screening tool that provides an estimate of global cognitive function, tapping aspects of attention, memory, language, and spatial abilities [32,33].

##### Attention/executive function

Trail Making Test A and B, Digit Symbol Coding, Letter Number Sequencing, and the Stroop Color Word Test were used to assess attention/executive function. Trail Making Test A requires participants to sequential order a series of numbers as quickly as possible and it is a reliable and valid measure of complex visual scanning and psychomotor speed [34]. Trail Making Test B asks individuals to quickly connect alternating numbers and letters [35]. Test completion time is a widely and valid used measure of executive function [34,36]. Digit Symbol Coding is a reliable and valid measure of visuomotor speed and complex attention and asks individuals to identify shapes that correspond with numbers as fast as possible [34,37]. Letter-number sequencing is a measure of working memory that involves verbally ordering numbers and letters that are orally presented in an unordered sequence. This task has excellent psychometric properties, with test-retest reliability of .75 [37]. Lastly, the Stroop Color Word Test [38] is a reliable measure that assesses executive function by examining an individual’s ability to inhibit a response [39,40].

##### Memory

The California Verbal Learning Test-II (CVLT-II) total recall, short delay free recall, and long delay free recall was used to assess memory function [41]. Individuals are asked to learn and recall a 16-item verbal word list. It is a reliable and widely used measure of verbal memory [41].

##### Fine motor dexterity

The Grooved Pegboard is a reliable measure used to assess fine motor dexterity [34,42]. Participants are asked to orient a series of grooved shaped pegs into corresponding holes in the pegboard as quickly as they can.

### Procedures

The Kent State University and Summa Health System Institutional Review Board (IRB) approved the study procedures and all participants provided written informed consent prior to study enrollment. The investigation conforms with principles outlined in the Declaration of Helsinki. As part of the larger NIH study procedures, a medical record review was performed and participants completed demographic and medical history self-report measures. A brief neuropsychological test battery was also administered to all HF patients as part of the larger study protocol to assess cognitive function. Upon completion of cognitive testing, HF participants were then recruited to complete a single time, 45-minute, driving assessment using the driving simulation technology.

### Statistical analyses

Two cases exhibited missing data for LVEF and those values were handled using simple mean imputation. For descriptive purposes and to facilitate clinical interpretation of cognitive test performance in the sample, raw scores for the neuropsychological test measures were transformed to T-scores (a distribution with a mean of 50 and standard deviation of 10) using normative data adjusting for age, and in the case of memory, gender. A T-score cutoff of ≤ 35 was used to characterize impairment in the cognitive domains. This cutoff was chosen because it is reflective of performances 1.5 SD below the mean relative to normative standards and it is a commonly used cutoff for the clinical identification and diagnosis of cognitive impairment [43].

For the main analyses, three separate composite scores were computed for attention/executive function, memory, and motor function that consisted of the mean of the raw scores of neuropsychological measures within each cognitive domain. To maintain directionality for attention/executive, scores for neuropsychological tests measured in units of time (i.e., Trail Making Test A and B) were multiplied by -1 so that lower scores reflect worse performance. Independent samples *t*-tests first examined driving simulation performance in this sample of HF participants compared to performance among healthy adults from a validation study [29] on the following indices: Total number of collisions, number of stop signs missed, number of centerline crossings, and number of road excursions.

A series of partial correlations adjusting for age, gender (1 = males; 2 = females), education, and LVEF were then conducted to examine the relationship between attention/executive function, memory, and motor function with the specific driving indices, including total collisions, number of stop signs missed, number of centerline crossings, number of road excursions, % of time over the speed limit, and % of time out of the lane.

## Results

### Cognitive function

The sample exhibited an average MMSE score of 27.83 (SD = 2.09). Specifically, 27.8% of the sample scored below a 27 and 11.1% scored below an MMSE score of 25. When using a T-score cutoff of ≤ 35 to characterize impairment in attention/executive function, memory, and motor functioning, 5.6% of the sample exhibited impairments in attention/executive function, 11.1% for memory, and 16.7% showed impairments in motor function.

Reduced performance on the driving simulation was also prevalent with many HF participants recording at least one collision (33.3%; max = 5). Similarly, HF participants also frequently crossed the centerline and deviated from the lane and speed limit on several occasions. Refer to Table 1 for a full summary of driving simulation and cognitive test performance.

### HF vs. healthy young adults on simulated driving performance

Independent samples *t*-test was conducted to examine driving simulation performance between the HF participants and healthy young adults. Independent samples *t*-test revealed that HF participants performed significantly worse than the healthy young adults on all of the driving simulation indices examined: Number of collisions (*t*(113) = -2.40, *p* = .02; M (SD) = 2.00(1.28) versus 1.30 (1.11)), number of stop signs missed (*t*(113) = -2.14, *p* = .045; M(SD) = .78(.81) versus .35(.60)), number of centerline crossings (*t*(113) = -2.40, *p* = .05; M(SD) = 5.83(3.96) versus 3.82(3.13)), and number of road excursions (*t*(113) = -2.44, *p* = .03; M(SD) = 4.83(6.39) versus 1.12(1.93)).

### Cognitive function and driving simulation performance

Partial correlations adjusting for age, gender, education and LVEF revealed significant associations between attention/executive function with number of centerline crossings (*r*(12) = -.56, *p* = .04) and % of time out of lane (*r*(12) = -.54, *p* = .045). In each, case worse attention/executive function was associated with reduced driving ability in each of these indices. A similar pattern also emerged for motor function: total number of collisions (*r*(12) = .57, *p* = .04), % of time over the speed limit (*r*(12) = .65, *p* = .01), and a trend for number of stop signs missed (*r*(12) = .47, *p* = .09). There was also a trend between reduced MMSE scores and greater number of stop signs missed (*r*(12) = -.50, *p* = .07). Memory performance demonstrated no associations with any of the driving indices. See Table 2.

**Table 2:** **Partial correlations between cognitive function and driving simulation performance (*N*** **= 18)**

**Driving indices**	**MMSE**	**Attention/executive function**	**Memory**	**Motor**
Total collisions	.15	-.35	.38	.57*
Stop signs missed	-.50Ψ	-.30	.25	.47Ψ
Centerline crossings	-.18	-.56*	.05	.41
Road excursions	-.01	.18	-.11	-.30
% Time over speed	.01	-.26	.27	.65*
% Time out of lane	.17	-.54*	-.06	.40

Note. *p < .05; Ψp < .10.

Partial correlations are adjusted for age, sex, education, and left ventricular ejection fraction.

## Discussion

Consistent with past work, reduced performance on cognitive testing was prevalent in this sample of HF patients. Extant evidence links cognitive impairment in HF with poor psychosocial outcomes (e.g., mortality) [3] and reduced ability to maintain functional independence, including tasks such as medication management and shopping [5,44]. The current study is the first to show that cognitive function is also associated with reduced performance on a simulated driving task in HF patients. Several aspects of these findings warrant brief discussion.

We found that HF participants performed significantly worse than healthy adults on the simulated driving scenario and that reduced attention/executive and motor function were associated with poorer driving simulation performance. Specifically, attention/executive function correlated with lane deviations and centerline crossings while motor function was associated with errors that require motor coordination (e.g., avoiding collisions). Consistent evidence shows that executive function is a key cognitive process required for driving ability in dementia populations [28]. On-road driving abilities require complex cognitive abilities (e.g., executive function) such as planning, multi-tasking, decision-making and awareness of driving environment [8]. Indeed, executive functions are consistent with multiple aspects of successful driving, including choosing the right travel route, estimating travel time, making good decisions, and adapting driving behaviors [17]. Reduced motor function has also been linked with increased driving errors in past studies, which is not surprising as motor coordination is required for steering accuracy and reaction time [45-48]. Future work should further explore how impairments in different cognitive domains in HF (e.g., attention/executive function) correspond to impairments in specific driving skills.

This pattern of findings is concerning, as the multiple brain regions (e.g., frontal, temporal, parietal lobes) responsible for these cognitive processes are susceptible to damage in HF patients as a result of ischemia [49]. Thus, such pathological changes may place HF patients at risk for poor driving performance [12,13,15]. For example, reduced performance on neuropsychological tests of executive function has been shown to predict unsafe driving and failure to pass on-road driving tests in patients with dementia [50]. Consistent with this pattern, elderly drivers with mild to moderate dementia are up to 8× greater risk of crashing than non-demented elderly [51]. If replicated, our findings encourage investigating the importance of driving assessment and clinical intervention (i.e. advise to discontinue driving) in this population.

Interestingly, the current study found no association between memory testing and simulated driving performance. The literature is inconsistent regarding the effects of memory on driving ability [52,53]. It is possible that memory impairments in cognitively impaired patients most likely translate to increased risk of becoming lost [54] rather than actual driving abilities. For example, past work that utilizes on-road testing shows that memory impairment in Alzheimer’s disease drivers is a strong predictor of route-following tasks further suggesting that memory plays a key role in a person’s ability to monitor and maintain orientation [55]. Future work that examines driving performance using on-road testing in HF is needed to determine whether memory impairments predict driving ability in this population.

Taken together, the association between driving simulation performance with frontal systems function, but not memory, is consistent with the extant evidence in HF that demonstrates executive function to be the most important cognitive ability for functional independence in this population [56]. It is possible that memory loss may be a better predictor of other outcomes in HF beyond the realm of functional capabilities such as mortality risk [57]. For instance, as suggested by the current study, even mild deficits in executive function may correspond to functional impairments such as reduced driving performance. Functional deficits associated with memory worsening may not occur until the more severe stages of HF and future work should explore this possibility.

The generalizability of the current findings is limited in several ways. First, cross-sectional data was analyzed and prospective studies are needed to confirm the link between cognitive decline and reduced driving ability in HF patients. Additionally, although simulated driving offers many advantages such as cost-effective and maintains the safety of the participant and the larger community [52,58], future work examining driving performance in HF patients using on-road testing would increase the ecological validity of the present findings. Future work is also needed in larger HF samples that also utilize age matched healthy elderly to confirm that cognitive dysfunction places HF patients at greater risk for reduced driving ability. Comparison of driving performance between HF patients and a healthy older adult control group would also permit inferences regarding the unique deficits (cognitive or otherwise) introduced in HF that increase risk for driving impairment in this population that extends beyond those associated with the normal aging process. Consistent with this notion, larger and more diverse samples of HF would also help clarify the relationship between the degree of cognitive impairment and driving performance. For instance, the current study failed to find a significant relationship between some of the cognitive domains and certain driving indices (e.g., executive function and collisions) and this may be reflective of range restriction on the cognitive test measures. Finally, although medical comorbidity and prescribed cardiac medications were prevalent in this sample of HF patients, the small sample size precluded inclusion of these factors as covariates. In-turn, future work should examine whether extensive medical and clinical comorbidities (including medication side effects) further exacerbate reduced driving ability in this population through there effects on cognition.

## Conclusions

In brief summary, the current study found that HF patients performed worse on a driving simulation scenario than healthy adults and reduced cognitive function was associated with poorer driving simulation performance in this sample. Such findings suggest that HF patients may be at-risk for driving impairment. Implementation of brief cognitive screening in clinical care settings may help to identify HF patients that may be unsafe for driving and thus allowing for earlier clinical intervention, including referral for a formal driving evaluation or advisement to family members to monitor patients’ driving. Future studies examining driving performance in HF patients using on-road testing are needed to confirm these findings and determine the most effective approach for identification of driving impairment in clinical settings.

## Competing interests

The authors declare no competing interests.

## Authors’ contributions

Study concept and design: MA, MBS, JG. Data acquisition: MA, MJC, JG. Analysis and interpretation of data: MA, JG. Manuscript preparation: MA, MBS, MJS, JG. All authors read and approved the final manuscript.

## Pre-publication history

The pre-publication history for this paper can be accessed here:

http://www.biomedcentral.com/1471-2318/13/58/prepub
